# Adapting to a new home: resettlement and mental health service experiences of immigrant and refugee youth in Montreal

**DOI:** 10.1057/s41599-023-01572-7

**Published:** 2023-03-07

**Authors:** Charles Gyan, Farhin Chowdhury, Ata Senior Yeboah

**Affiliations:** 1 School of Social Work, McGill University, Montréal, QC Canada; 2 Department of Education and Counselling Psychology, McGill University, Montreal, QC Canada

**Keywords:** Sociology, Social policy

## Abstract

Resettlement and mental health services are critical support systems for refugee and immigrant youth (RIY) as they navigate the complexities of settling into their new homes. These services play a vital role in meeting the needs of RIY and helping them feel welcomed into Canadian society. The purpose of this study is to provide insight into RIY’s experiences with resettlement and mental health service providers in Montreal, Canada. Adopting a descriptive quantitative research approach, this study utilized online surveys to gather data. The findings indicate that cultural and linguistic barriers are the major obstacles faced by refugee and immigrant youth when accessing resettlement and mental health services in Montreal. Protective resources, such as family, friends, and ethnic communities, were identified as important facilitators of successful integration into Canadian society. To improve services, cultural sensitivity should be a priority for providers, as recommended by this study. By acknowledging the significance of cultural barriers in accessing resettlement and mental health services, this study emphasizes the need for service providers to prioritize cultural sensitivity in their efforts to improve services.

## Introduction

Canada’s rich history of embracing immigrants has helped shape it into a vibrant mosaic of diverse cultures, making it truly a “nation of immigrants” that cherishes and celebrates multiculturalism. Between 2011 and 2016, Canada welcomed over 1.2 new immigrants, representing 21.9% of the total population in Canada (Statistics Canada 2017). According to the 2016 census, more than one in five Canadians are born outside of Canada and this foreign-born population is projected to reach between 24.5% and 30% by 2036 (Statistics Canada 2017). As the world was gripped by the pandemic, the influx of new immigrants into Canada dwindled significantly in 2020. This was due to the harsh realities brought about by the global health crisis such as the shutting down of offices, closed borders, and other restrictions aimed at containing the spread of the virus. As a result, the proportion of population growth attributed to immigration plummeted from three-quarters to a modest 58% (Immigration, Refugees, and Citizenship Canada (2022)). Since then, new measures have been implemented to attract newcomers to Canada and mitigate the impacts of COVID-19 (Immigration, Refugees, and Citizenship Canada (2022)). Nevertheless, immigration has significantly shaped Canada and continues to play a vital role in helping the country fuel its economic growth, especially by contributing towards the post-pandemic economic recovery (Immigration, Refugees, and Citizenship Canada (2022)).

Canada has primarily admitted immigrants under the economic category with the objective of promoting economic growth (Statistics Canada 2017). In addition, refugees have also made up a significant portion of Canada’s immigrant population, seeking asylum from conflicts, famine, and economic instability (Guruge and Butt 2015; Statistics Canada 2017). Global conflicts and climate change have forced families and children to flee their homes and settled in foreign countries (see for e.g., Levy 2019; Reuveny 2007). Due to the humanitarian crisis and as part of Canada’s commitment to contribute to global refugee protection, the country plans to welcome 40,000 at-risk Afghan refugees and support their safe resettlement (Immigration, Refugees, and Citizenship Canada (2022)).

With this increasing number of newcomers, the vast majority reside in Montreal, one of the largest metropolitan areas in Canada. While the city has attracted immigrants from various parts of the world, a significant proportion comes from Asian (such as India, China, and Syria) and African countries (like Nigeria, Algeria, and Egypt) (Statistics Canada 2017). Although diversity is a fundamental aspect of Canadian national identity, discrimination and racism are still prevalent, especially among people of color (Rossiter et al. 2015). Due to these and other challenges, the journey of RIY has not been a smooth one. They face unique challenges that affect their settlement and adaptation experiences. For instance, among the multiple challenges and barriers encountered by newcomers, youth report facing socioeconomic hardships, challenging educational experiences, marginalization, language barriers, and cultural identity issues (Rossiter et al. 2015; Smith et al. 2022). However, beyond the hardships, many others have encountered opportunities and successes as they aim to better their lives and that of their families in their new country. Their positive experiences can be explained in part by their strong social support system, community involvement, and connection with home culture and religion (Rossiter et al. 2015; Smith et al. 2022). Furthermore, in effort to make their transitions easier, settlement service providers and mental health providers have been working with newcomer youth as they navigate their settlement process. These services play an important role in meeting their various needs, including providing support to integrate successfully and making them feel welcomed in Canadian society (Immigration, Refugees, and Citizenship Canada (2022)). Mental health services become particularly important for newcomer youth as they attempt to navigate a new culture and language while going through developmental changes (e.g., puberty, identity formation) in this period of life (Juang et al. 2018; Smith et al. 2022).

Despite exhibiting remarkable resilience and often enjoying better physical and mental health than their Canadian-born peers, immigrant youth are not immune to the mental health challenges stemming from acculturation stress, discrimination, social isolation, and premigration trauma such as war, famine, and natural disasters (Guruge and Butt 2015; Pumariega et al. 2005; Smith et al. 2022). The resettlement stress has been related to difficulties in accessing proper resources such as resettlement services and health care which can exacerbate mental health concerns as newcomers attempt to navigate the new and unfamiliar system (Ellis et al. 2020). Immigrant youth may face unique challenges in the resettlement process that can have a significant impact on their mental health outcomes. By tailoring support services to the unique needs of these youth and providing culturally responsive care, we can help mitigate some of these risks and promote positive resettlement experiences. As noted by Guruge & Butt (2015) and Pumariega et al. (2005), appropriate and individualized support can play a critical role in supporting the mental health and well-being of immigrant youth and ensuring that they are able to thrive in their new communities. A critical function of social and mental services is fostering social connectedness to reduce feelings of isolation (Smith et al. 2022). By helping immigrant youth feel connected to their communities, mental health services can promote resilience and reduce the risk of mental health challenges. To facilitate the healthy development of immigrant youth, it is essential that they have sufficient access to services and resources that can help them overcome the barriers associated with adjusting to a new culture. Such services and resources can also promote community resilience and foster a sense of belonging, which is crucial for their overall well-being (Smith et al. 2022). Despite the risks and challenges experienced by newcomer youth, there is a lack of understanding on whether these youth are getting the adequate support during their resettlement process and whether their needs are being met or otherwise. As such, this study seeks to understand the different barriers and facilitators faced by refugee and immigrant youth by examining their experiences with resettlement and mental health services in Montreal.

## Literature review

Canada, a country that owes much of its growth and success to immigration, has experienced a substantial increase in the population of immigrants and refugees in recent years (Smith et al. 2022; Statistics Canada 2017). Among the Canadian cities that have welcomed newcomers, Montreal stands out as a thriving hub of ecocultural diversity. However, successful adaptation to a new country remains a common challenge for many immigrants and refugees, particularly for youth, who face unique obstacles to their healthy development (Rossiter et al. 2015; Smith et al. 2022). Given the complexity of the resettlement process and the associated mental health challenges, it is imperative to pay attention to the experiences of immigrant and refugee youth in Montreal. To better support, these young people, a more comprehensive understanding of their access to mental health services and their interactions with these services are needed. Through such an inquiry, we can gain insight into the resources that are most effective in promoting positive mental health outcomes for immigrant and refugee youth and how we can best support them in the long run.

## Mental health of newcomer youth

Emerging adulthood, a new life stage between the ages of 18 and 29, is an important transition in their life and it is characterized by a period of heightened instability as they experience important changes in their life (e.g., relationships, jobs) (Arnett et al. 2014). This is particularly true for young immigrants building a new life in a different country and trying to make sense of their new land. Their mental health during this time has important implications as they go through identity formation, uncertainties, and feelings of optimism (Arnett et al. 2014). The changes and instability may take a toll on their mental health, especially if they find themselves in a new environment without adequate social support (Arnett et al. 2014; Simich et al. 2005). In fact, previous studies have shown that although immigrants tend to have better psychological well-being compared to the native-born upon arrival in Canada, their mental health may deteriorate with time (Vang et al. 2017). This phenomenon is known as the “healthy immigrant effect” and can be explained by the immigration healthy policy and systemic barriers that jeopardize their health advantage (Beiser 2005; Thompson et al. 2015; Vang et al. 2017). The mental health disparities may be explained by numerous factors related to their resettlement experiences. Studies have previously found that immigrant youth are at risk of experiencing high levels of stress and mental health challenges due to their immigrant settlement situation such as cultural assimilation and difficulties adapting to a new environment (Beiser 2005; Shields and Lujan 2018). The mental health challenges, such as high level of emotional distress, including depression and symptoms of post-traumatic disorder can also be related to refugee and other immigrant youths’ premigration traumatic experiences such as exposure to political violence and losses or separations (Beiser 2005; Rousseau et al. 2004; Weine 2008). Despite the mental health complications, many young adults decide not to seek support from mental health providers (Arnett et al. 2014; Tiwari and Wang 2008). The pervasive underutilization of mental health services among immigrants and refugees can be attributed to multifaceted and complex barriers that hinder their access to care (Ellis et al. 2020; Thompson et al. 2015). These barriers include a lack of awareness of available services, cultural and linguistic mismatches between providers and clients, insufficient financial resources, negative settlement experience (i.e., social isolation, financial insecurity, discrimination), and the stigma surrounding mental illness, among others (Ellis et al. 2020; Simich et al. 2005; Thompson et al. 2015). Thus, a deeper look into refugee and immigrant youth experiences with mental health services and resettlement are warranted.

## Resettlement experiences

Refugees and immigrants are often resettled in low-resource communities and in neighborhoods with higher rates of crime and violence, which has an impact on their health (Beiser 2005; Ellis et al. 2020). The resettlement process presents a multitude of challenges, including but not limited to inadequate access to healthcare, financial instability, and insufficient resources to meet basic needs (Ellis et al. 2020; Simich et al. 2005). Specifically, immigrant youth in Canada are likely to experience high levels of unemployment, face barriers to their educational attainment as they take on adult responsibilities (e.g., the breadwinner of the family), and encounter inadequate and culturally inappropriate social services (Shields and Lujan 2018). The challenges associated with resettlement have a disproportionate impact on immigrants, as they often have limited resources compared to native-born individuals (Beiser 2005). In the context of migration, individual stress (e.g., premigration trauma), family stress (e.g., family conflict), school stress (e.g., peer bullying), and community stress (e.g., lack of belongingness) play a role in youth resettlement experience (Juang et al. 2018). The resettlement process may be different for different newcomers based on various factors (e.g., age, socioeconomic status, country, or region), but typically isolation and loneliness are common due to experiences of discrimination and marginalization (Ellis et al. 2020). Prior studies have established a direct link between cultivating positive relationships with families and peers, engaging in community activities, and fostering a positive resettlement experience for youth (Ellis et al., 2020; Juang et al. 2018; Weine 2008). These factors have also been shown to act as a protective shield, mitigating the potential negative impacts of migration on young individuals (Ellis et al. 2020; Juang et al. 2018; Weine 2008). Further, active involvement in the cultural community and participation in cultural activities has been associated with positive ethnic identity and lower level of mental health complications (Shields and Lujan 2018; Simich et al. 2005). RIY place significant value on preserving their cultural identity and remaining authentic to themselves as a means of promoting resilience and a sense of purpose in their new Canadian home (Smith et al. 2022). Service providers may help promote their social adjustment and reduce the resettlement stress among these youth by capitalizing on these protective factors.

## Role of service providers

The availability of resettlement and mental health services can significantly impact the adjustment of newcomers to their new country. However, cultural differences and incompatibility can act as a barrier, hindering immigrants and refugees from accessing these vital services (Simich et al. 2005; Thompson et al. 2015). For example, some members of ethnic minority groups may believe that it is preferable to rely on families, religion, or “stay strong” while others may be reluctant to use such services due to the gender roles and stigma associated with seeking mental health services (Simich et al. 2005; Thompson et al. 2015). However, those who do seek mental health services may receive suboptimal care due to the cultural and language barriers (Thompson et al. 2015; Tiwari and Wang 2008). As a result, it is not surprising that many immigrants in Canada underutilize the mental health services. This underutilization of mental health services among immigrants highlights the pressing need for service providers to offer culturally responsive care that is tailored to the unique needs and circumstances of this population, be trained in working with multicultural clients, and hire more ethnically and linguistically diverse staff (Shields and Lujan 2018; Thompson et al. 2015).

The provision of resettlement and mental health services can play a crucial role in mitigating the adverse effects of migration and supporting immigrants during their transition to a host country. Importantly, it should be noted that many immigrants have been able to successfully integrate into their host countries thanks to the support provided by such services (Smith et al. 2022). Settlement service providers have a pivotal role to play in supporting the resettlement of refugee and immigrant youth. In addition to helping them connect with their community and establish supportive social networks, settlement service providers can offer a range of other services, such as assistance with employment and housing searches, as well as health and mental health services (Ellis et al. 2020; Weine 2008). These services can be instrumental in promoting a sense of belongingness and facilitating the integration of refugee and immigrant youth into their new communities (Ellis et al. 2020; Weine 2008). To further our understanding of the resettlement experiences of immigrant and refugee youth in Montreal, this study aims to investigate the extent to which their needs are being met by settlement service providers. Through a deeper understanding of the lived experiences of young immigrants and refugees, and identifying areas where service provision may fall short, we can endeavor to make settlement services more responsive and attuned to the needs of this population. This, in turn, can facilitate a smoother transition and integration into their host community, ultimately supporting their successful resettlement and fostering a sense of belonging.

## Methods and materials

We adopted descriptive quantitative research approach for the study given that the purpose of the research was to explore refugee and immigrant youth’s experiences with resettlement and mental health services in Montreal. An online survey was implemented from *January 2022 to April 2022* to gather data from refugee and immigrant youth in Montreal, Canada. Refugee and immigrant youth are better positioned to provide useful information about resettlement and mental health services in Montreal owing to their relative experiences and encounters with these service providers. We invited refugee and immigrant youth to participate in the survey through *social media (Facebook and WhatsApp).* Participation in the research was voluntary. RIYs that were interested contacted the principal investigator, and they were sent an electronic form of the survey questionnaire to be filled. The inclusion criteria in the recruitment of participants were refugee and immigrant youths within the ages of 15–29, newcomer youth that had lived in the city of Montreal for at least two years as well as youths that were proficient in English Language (oral and written). The research adapted the Child and Youth Resilience Measure (a validated resilience scale) put forward in the work of Ungar & Liebenberg (2011). The scale has been designed to measure child and youth development across different cultures, thus, making it appropriate for our study. The scale focuses on relational, individual, community and cultural dimensions of resilience (Ungar and Liebenberg 2011).

To operationalize the resilience scale, a Likert type questionnaire was developed and used in the study. The questionnaire covered constructs such as individual, relational, community and cultural resilience as well as experiences with resettlement and mental health services. The responses to the statements/questions were captured on a three-point scale (1 to 3), with 1 indicating for example disagreement and 3 indicating agreement to the statement or question. The other aspects of the survey questionnaire focused on the socio-demographic profile of participants such as age, educational level, and region of origin. These variables are key as they could influence the resilience of participants. Questions such as whether participants sought mental health support, how they coped with resettlement challenges without support from resettlement organizations, whether services provided were sensitive to the culture of respondents among others were posed to provide a profound understanding of RIYs experiences with resettlement and mental health services.

It is important to indicate that the survey was self-completed. We received responses from *110* refugee and immigrant youth. Following this, we checked for cases with over 10% of missing and/or inaccurate data for deletion. As suggested by Hair et al., (2014) data sets with more than 10% of missing/inaccurate data become prospective candidates for deletion. Having subjected the data sets to scrutiny, we found that *17* respondents did not fully answer the survey questions or had provided inaccurate responses. These *17* responses were accordingly deleted, thus, making us retain *93* responses as the focus of analysis for the present study. Table 1 provides a summary of the socio-demographic characteristics of the respondents.

**Table 1:** Socio-demographic characteristics of participants – nominal variables (*N* = 93).

	Frequency	Percent
Age (in years)		
18–20	12	12.9
21–23	54	58.1
24+	27	29.0
Region of Origin		
Middle East	15	17.2
Africa	19	21.8
Europe	8	9.2
Asia	14	16.1
Latin America	4	4.6
North America	27	31.1
Overall Resilience		
Low resilience	49	52.7
Moderate resilience	18	19.4
High resilience	14	15.1
Exceptional resilience	12	12.9
Personal resilience		
Low resilience	1	1.1
Moderate resilience	24	25.8
High resilience	43	46.2
Exceptional resilience	25	26.9
Relational Resilience in groups		
Low resilience	3	3.2
Moderate resilience	13	14.0
High resilience	41	44.1
Exceptional resilience	36	38.7

We applied descriptive analysis to explore the quantitative data. With the aid of descriptive quantitative data analysis, we were able to describe and identify pertinent trend in our data to help us achieve our research objectives. Further, with the aid of SPSS software (version 27), we undertook a descriptive analysis of participants levels of resilience as well as their experiences with resettlement and mental health services in Montreal.

## Results

### Sample description

Table 1 depicts participant’s demographic information. From Table 1, it is clear that most of the participants were between 21 and 23 years of age (58.1%, *n* = 54). Regarding the origin of the participants, North America constitute (31.1%, *n* = 27), Africa (21.8%, *n* = 19), and Middle East making up (17.2%, *n* = 15). With respect to respondents’ education level, nearly three-quarters (74.2%, *n* = 69) had more than a high school diploma, with the greatest share having a four-year degree (36.6%, *n* = 34). Also, it is evident that more than 50% of the participants acknowledged having a low level of resilience (52.7%, *n* = 49) when questioned about their overall level of resilience. However, for personal resilience, the results indicated that most respondents demonstrated a high level of resilience (46.2%, *n* = 43). A similar pattern emerged for the results related to relational resilience: 44.1% (*n* = 41) reported having high resilience, with another 38.7% (*n* = 36) expressing exceptional resilience.

### Experience with resettlement and mental health agencies in Montreal

With respect to the services that could be received, either by mental health professionals or resettlement agencies, Table 2 below shows the following: When asked if they would accept mental health support if they were upset for an extended period, participants responded with mixed opinions. Indeed, 39.8% (*n* = 37) were uncertain, compared to 43% (*n* = 40) who agreed with the statement. Moreover, it is evident that 45.2% (*n* = 42) of the surveyed participants felt that their family would best serve them in solving their problems, and therefore, seeking professional support would be a last resort. In a more outstanding way, respondents stated that they were able to effectively handle the resettlement challenges they encountered without the support of a resettlement organization (51.6%, *n* = 48). Consequently, most could not say if the support provided by settlement agencies and mental health services was sufficient to improve their personal coping strategies (45.2%, *n* = 42). About these coping strategies, only 34.4% (*n* = 32) participants felt that the strategies offered by the agencies conflicted with their cultural messages received by family and community. However, the results shown are quite close as those undecided about this statement represent 35.5% (*n* = 33) and participants disagreeing 30.1% (*n* = 28). Besides, most of the participants (39.8%, *n* = 37) were not sure that the services they received recognized and supported the positive cultural messages communicated by their family, friends, and community.

**Table 2:** Mental health and resettlement agencies programs (*N* = 93).

	Frequency	Percent
Sought mental health support if needed		
Disagree	16	17.2
Not Sure	37	39.8
Agree	40	43.0
Family as a mental health support before professional support		
Disagree	13	14.0
Not Sure	38	40.9
Agree	42	45.2
Coped with resettlement challenges without support from resettlement organizations		
Disagree	10	10.8
Not Sure	35	37.6
Agree	48	51.6
Settlement agencies and mental health services enhanced my coping strategies		
Disagree	24	25.8
Not Sure	42	45.2
Agree	27	29.0
Agencies’ strategies/support in conflict with cultural messages		
Disagree	28	30.1
Agree	32	69.9
Service providers respected my native language		
Disagree	29	31.2
Not Sure	31	33.3
Agree	33	35.5
Agencies connected immigrants to culturally or socially supportive organizations		
Disagree	51	55.5
Agree	41	44.5
Program reinforced basic core values shared by my family		
Disagree	39	41.9
Not Sure	27	29.0
Agree	27	29.0
Program taught skills relevant to one’s cultural and social networks		
Disagree	26	28.0
Not Sure	33	35.5
Agree	34	36.6
Satisfied with the resettlement services received in Montreal		
Disagree	59	64.1
Agree	33	35.9

The results of the study suggest that service providers showed varying degrees of respect for the native language or dialect of respondents during language instruction. The distribution of responses was roughly even, with approximately one-third of respondents falling into each category: 35.5% (*n* = 33) agreed, 33.3% (*n* = 31) were undecided and 31.2% (*n* = 29) disagreed. Nonetheless, participants (44.1%, *n* = 41) reported that the agencies connected them to culturally and socially supportive organizations and events in the community. Still from Table 2, it appears that less than 30% of participants surveyed viewed the program as reinforcing the core values shared by the participant’s family, with the largest proportion (41.9%, n = 39) feeling uncertain about this statement. A total of 36.6% (*n* = 34) participants believed that the program’s taught and modeled skills were relevant for themselves to interact well with their cultural and social networks. Overall, while most participants (38.7%, *n* = 36) did not know if they were satisfied with the resettlement services they received in Montreal, slightly more than 35% reported being satisfied (35.5%, *n* = 33).

## Discussion

Immigrant and refugee youth in Canada can face challenges during the resettlement process, and a better understanding of their experiences in Montreal is necessary to facilitate their settlement in their new home. Although resettlement services play an important role in the integration of newcomers (Smith et al. 2022), overall, our finding revealed mixed opinions regarding their satisfaction with the resettlement services among youth in Montreal. The uncertainties may arise due to cultural clash, as many of the youth have reported that the coping strategies and cultural message shared by the agencies did not align with the messages and values received by their family, friends, and community, and some have also reported lack of respect towards their native language. This is in line with previous studies suggesting that cultural insensitivity and incompatibility are one of the major barriers experienced by immigrants (Thompson et al. 2015). Although the findings from our study suggested that the Western health care system and resettlement agencies can fail to consider the cultural worldview of the newcomers, the young adults felt that these agencies could connect them to their cultural events and communities. Maintaining socio-cultural ties with members of the community is important to their cultural identity and sense of belonging (Shields and Lujan 2018; Simich et al. 2005), especially since the social network can be disrupted or weakened when arriving in a new country (Arnett et al. 2014; Stewart et al. 2008).

In the literature, it has been established that immigrant and refugee youth underutilize mental health services compared to their Canadian-born counterpart (Thompson et al. 2015; Tiwari and Wang 2008). Our finding suggested that while many newcomers are willing to seek mental health services, many are also uncertain. The uncertainties could be explained by various reasons, including the language barriers and discrimination they face (Rossiter et al. 2015; Smith et al. 2022). Additionally, seeking mental health support varies across culture, age, gender, and socioeconomic statuses (Thompson et al., 2015; Tiwari & Wang, 2008), which could explain the variations in the participants’ answers when asked about their opinion on whether they would seek mental health support. For example, immigrant seniors and/or women can resist mental health support due to the stigma associated with mental health issues (Thompson et al. 2015). As such, because of the stigma and cultural beliefs, there may be a reluctance to seek outside help and confer to families and friends for support and to alleviate mental health-related symptoms such as stress (Thompson et al. 2015). In fact, our studies showed that immigrant and refugee young adults preferred relying on families rather than professional support.

Families and parental involvement have been identified as a protective factor in the scientific literature on newcomers and mental health, especially as young adults face stressful life transitions as they migrate (Simich et al. 2003; Wong et al. 2007). Having a social support system have been linked to better well-being among immigrants and refugees (Simich et al. 2003; Wong et al. 2007). As such, immigrants may be more likely to use their social support network to cope with problems rather than seeking professional support. Family support remains a dominant and important value in many cultures, especially among collectivist and interdependent, as they can provide various types of support, including encouragement and emotional support (e.g., Wong et al. 2007), as well as informational support (e.g., Simich et al. 2003). For example, a study conducted with refugees in Canada revealed that settlement service providers and immigration officials provided limited information and that the information provided was often inaccurate, which resulted in refugees relying on others with similar experiences (Simich et al. 2003). Another study with immigrant and refugees indicated that, for individuals from a collectivist culture, families and friends were major sources of support for Somali and Chinese newcomers in Canada, and some even seek support from informal sources because of the discrimination experienced when seeking professional support (Stewart et al. 2008). In this vein, although, professional services may provide emotional and informational support, immigrant and refugee youth may lean towards seeking support from more intimate sources such as their friends and families to meet their needs given the shared experience and understanding (Simich et al. 2003).

In addition, the young people in our study reported relying on their own resources to handle the resettlement challenges without the support of resettlement organizations. As important it is to study the challenges, it is equally important to attend to the resilience factors that can contribute to newcomers’ adjustments (Smith et al. 2022). Participants in our study showed high levels of personal and relational resilience. Previous research has revealed that many newcomer youths show resiliency by maintaining a connection with home culture and religion, positive thinking, and receiving emotional support (Motti-Stefanidi and Masten 2020; Smith et al. 2022). As such, our finding may suggest that many young newcomers are resilient as they can capitalize on their strengths and own resources to handle the challenges without the support of organizations. That is, despite the challenges they may face, they may draw on personal or relational resources to overcome the difficulties associated with the resettlement. This highlights the importance of focusing on how newcomers can withstand adversity and demonstrate resilience.

The results of our study paint a complex picture of the integration experiences of refugee and immigrant youth. On the one hand, the findings underscore the importance of social and cultural ties in facilitating a sense of belonging and community connectedness. The support of family and friends, as well as the larger community, was shown to be instrumental in promoting successful integration. On the other hand, the study revealed that several factors can pose significant challenges to the integration process. These include discrimination, agency approaches that do not account for cultural differences, and a lack of respect for the unique language and cultural backgrounds of newcomers. Addressing these potential barriers will require a concerted effort from all stakeholders involved in the resettlement process, including service providers, community leaders, and policy makers. Only through a collaborative, asset-based approach that values the experiences and perspectives of refugee and immigrant youth can we foster a more inclusive and welcoming society for all.

### Limitation and future directions

There is a need for research to further understand the barriers and facilitators faced by adult newcomers in Montreal. Given that resettlement service uses, and experiences can be influenced by demographic characteristics, future studies can employ sensitivity analyses to differentiate the experiences among various newcomer populations in Montreal. It should be noted that, although participants in our study reported a variety of ethnicity, the findings may not be fully generalizable as participants were predominantly North American. The findings from this study are to be interpreted with caution as Montreal is known for providing multilingual services and the experiences with resettlement services may be different in different geographical locations.

One potential limitation of this study is that it relies solely on self-reported data. Future research that includes multiple informants’ reports could help address possible recall and reporting biases. Additionally, the unique cultural and national identities of the refugee and immigrant youth participants could impact their resettlement experiences and interactions with mental health services in Montreal. While we acknowledge this as a limitation of the study, we encourage readers to interpret our results and findings with this in mind.

It is also important to note that, due to the cross-sectional nature of this study, we cannot establish causal relationships between variables. Nonetheless, our findings highlight critical implications for service providers to better understand the resettlement experiences of refugee and immigrant youth and deliver culturally appropriate services that are sensitive to their unique values, language, and strengths (Thompson et al. 2015).

## Conclusion

The steady influx of newcomers to Canada necessitates a thorough understanding of the barriers they face in adapting to their new home. With this in mind, our study aimed to explore the resettlement and mental health experiences of immigrant and refugee youth in Montreal, Canada. The findings of the study illuminate some of the challenges immigrant and refugee youth encounter in Montreal, including linguistic and cultural differences, as well as the potential sources of resilience they possess, such as their personal resources, ethnic community, and family support. This study highlights the need for resettlement service providers to leverage these protective resources to facilitate successful adaptation and integration of RIY into their host communities. Moreover, recognizing the significance of cultural identity and familial bonds, policies and programs should strive to cultivate a culturally sensitive approach that acknowledges the needs of young adult newcomers in Montreal. This study not only sheds light on the multifaceted and ever-evolving experiences of RIY, but also advocates for the creation of culturally responsive and inclusive environments in their host country that foster a sense of connection and belonging. By acknowledging and dismantling the systemic barriers that impede the successful integration of RIY, we strive to forge a pathway to a seamless transition and positive resettlement experience in their new home.

## Data Availability

The datasets generated during and/or analysed during the current study are available from the corresponding author on reasonable request.
